# Therapeutic Efficacy and Macrofilaricidal Activity of Doxycycline for the Treatment of River Blindness

**DOI:** 10.1093/cid/ciu1152

**Published:** 2014-12-23

**Authors:** Martin Walker, Sabine Specht, Thomas S. Churcher, Achim Hoerauf, Mark J. Taylor, María-Gloria Basáñez

**Affiliations:** 1 Department of Infectious Disease Epidemiology, Imperial College London, United Kingdom; 2 Institute for Medical Microbiology, Immunology and Parasitology, University Hospital Bonn, Germany; 3 Department of Parasitology, Liverpool School of Tropical Medicine, United Kingdom

**Keywords:** onchocerciasis, doxycycline, macrofilaricide, efficacy, clinical trials

## Abstract

***Background.*** *Onchocerca volvulus* and lymphatic filariae, causing river blindness and elephantiasis, depend on endosymbiotic *Wolbachia* bacteria for growth, development, fertility, and survival. Clinical trials have shown that doxycycline treatment eliminates *Wolbachia*, causing long-term sterilization of adult female filariae and effecting potent macrofilaricidal activity. The continual reinfection by drug-naive worms that occurs in these trial settings dilutes observable anti-*Wolbachia* and antifilarial effects, making it difficult to estimate therapeutic efficacy and compare different doxycycline regimens, evaluated at different times after treatment.

***Methods.*** A meta-analytical modeling framework is developed to link all usable data collected from clinical trials measuring the *Wolbachia* status and viability of individual female adult worms collected at various times after treatment with 4, 5, or 6 weeks of daily 100 or 200 mg oral doxycycline. The framework is used to estimate efficacy parameters that are not directly measurable as trial outcomes.

***Results.*** The estimated efficacy of doxycycline (the maximum proportional reduction in the percentage of adult female *O. volvulus* positive for *Wolbachia*) is 91%–94% on average, irrespective of the treatment regimen. Efficacy is >95% in the majority of trial participants. The life span of *Wolbachia*-depleted worms is reduced by 70%–80%, from approximately 10 years to 2–3 years.

***Conclusions.*** The efficacy parameters are pertinent to the prospects of using doxycycline on a “test and treat” basis for onchocerciasis control and confirm doxycycline as a potent macrofilaricidal therapy. The modeling approach is more generally relevant to the design and evaluation of clinical trials for antifilarial drugs conducted in endemic settings.

## Abstract (precis)

The efficacy of 4–6 weeks of oral doxycycline in depleting *Wolbachia* from *Onchocerca volvulus* is >95% in the majority of patients. *Wolbachia* depletion induces a 70%–80% reduction in worm life span, confirming doxycycline as a potent macrofilaricide.

Most filarial parasites of humans depend on endosymbiotic *Wolbachia* bacteria for growth, development, fertility, and survival [1]. Clinical trials have shown that eliminating *Wolbachia* from *Onchocerca volvulus* and *Wuchereria bancrofti*—the etiological agents of onchocerciasis (river blindness) and (bancroftian) lymphatic filariasis (elephantiasis), respectively—with a course of oral doxycycline permanently sterilizes female worms and reduces the adult worm longevity, inducing potent macrofilaricidal activity (ie, the killing of adult [macro]filariae). These properties are superior to those of other currently used antifilarial drugs—diethylcarbamazine, ivermectin, and albendazole—which predominantly act against the microfilarial progeny of female worms, having somewhat limited activity against adults [2]. This particularly applies to onchocerciasis, for which only ivermectin is used for mass drug administration.

Clinical trials of anti-*Wolbachia* therapy have typically evaluated antifilarial activity using immunohistological, molecular, and morphological criteria, measured at various times after treatment. In lymphatic filariasis, loss of female fertility is demonstrated through absence of microfilaremia (microfilariae in the blood), whereas worm viability is assessed by loss of ultrasonographic detection of “filarial dance sign” (a pathognomonic type of movement by live adult worms within worm nests) and reduction in circulating filarial antigenemia [3]. In onchocerciasis, worm fertility and viability are determined by immunohistological and morphological examination of *O. volvulus* macrofilariae within subcutaneous nodules excised from trial participants [4]. Female fertility is monitored by reduced microfilaridermia (microfilariae in the skin) [5].

The dynamics of the antifilarial outcomes induced by doxycycline are protracted because of the indirect mode of action of anti-*Wolbachia* therapy on parasites' vital processes. Doxycycline elicits a gradual yet sustained reduction in microfilaridermia, not because it kills *O. volvulus* microfilariae directly, but because the microfilarial population is not replenished by (sterilized) female worms, with skin microfilariae declining through natural attrition. Adult worms suffer a similar “slow-kill” effect. In *O. volvulus*, an increased abundance of dead worms is observed approximately 2 years after the start of doxycycline therapy. In *W. bancrofti*, an increase in the proportion of patients without detectable worm nests (loss of filarial dance sign) is observed between 12 and 18 months [6].

The slow antifilarial activity of anti-*Wolbachia* therapy confers an excellent safety profile by avoiding inflammatory reactions associated with rapid killing of micro- or macrofilariae [2]. However, it also complicates interpretation of antifilarial outcomes; during a long follow-up period, trial participants, who continue to live in endemic areas with ongoing transmission, become reinfected with worms harboring a full complement of *Wolbachia*. This dilutes the apparent antifilarial efficacy of a treatment regimen [7]. Furthermore, the intrinsic time dependency of antifilarial activity makes it difficult to compare outcomes measured after different follow-up periods, in different epidemiological settings, from patients treated for different durations with different doses.

Modeling the interacting temporal dynamics of *Wolbachia* depletion, antifilarial activity and parasite infection can resolve these issues by providing a framework with which to link data from clinical trials that used different treatment regimens and follow-up periods. Taking this approach, we (1) estimate and compare the efficacy of different treatment regimens in eliminating *Wolbachia* from female worms, and (2) quantify the ensuing macrofilaricidal activity in terms of a reduced adult life span. We discuss our results in the context of alternative or complementary interventions required to achieve proposed control and elimination goals.

## METHODS

### Data

The data originate from 1 randomized, placebo-controlled trial [8] and 2 open (nonrandomized, untreated controls) trials [9, 10] on the effects of doxycycline on *O. volvulus*, the only field trials to have collected useable data on individual adult *O. volvulus* (Supplementary Methods, Systematic Review). Participants infected with *O. volvulus* micro- and macrofilariae received 4, 5, or 6 weeks of directly observed 100 or 200 mg oral doxycycline (or placebo) daily and were followed up on multiple occasions to have operable onchocercomas extirpated. Participants not completing the course of treatment were not followed up (Supplementary Methods, Participant Compliance and Follow-up). Each *O. volvulus* was categorized as dead or alive, and conditional on being alive, *Wolbachia* positive or *Wolbachia* negative by morphological and immunohistological analyses. The data are summarized in Table 1 and Supplementary Table 1. Some participants received ivermectin either as part of study protocols or prior to receiving doxycycline (mainly from mass drug administration programs [8, 9, 10]). Such data are not included in the analysis because ivermectin does not affect *Wolbachia* [8, 9] nor (at recommended regimens [11]) the longevity of adult *O. volvulus*.

**Table 1.** Summary of Nodulectomy Data Collated From 3 Clinical Trials on the Effects of Doxycycline on Female *Onchocerca volvulus*

Regimen		Follow-up, mo After Treatment	Participants	Extirpated Nodules	Female Worms by *Wolbachia* Status		Dead Female Worms
Duration, wk	Dose, mg, per day				Positive	Negative	
4	200	20–39^a^	14	72	17	42	62
5	100	20–27	20	96	20	63	79
6	100	2–18	62	217	72	321	91
6	200	6–39^a^	18	115	27	71	109
6	Placebo	5.5–27	23	103	150	10	46
Untreated	NA	NA	45	142	236	8	44

Abbreviation: NA, not applicable.

^a^ Data from patients nodulectomized at the 39-month follow-up time were not presented as part of the main analysis in the original study [8].

### Population Dynamics Model

**Figure 1.**
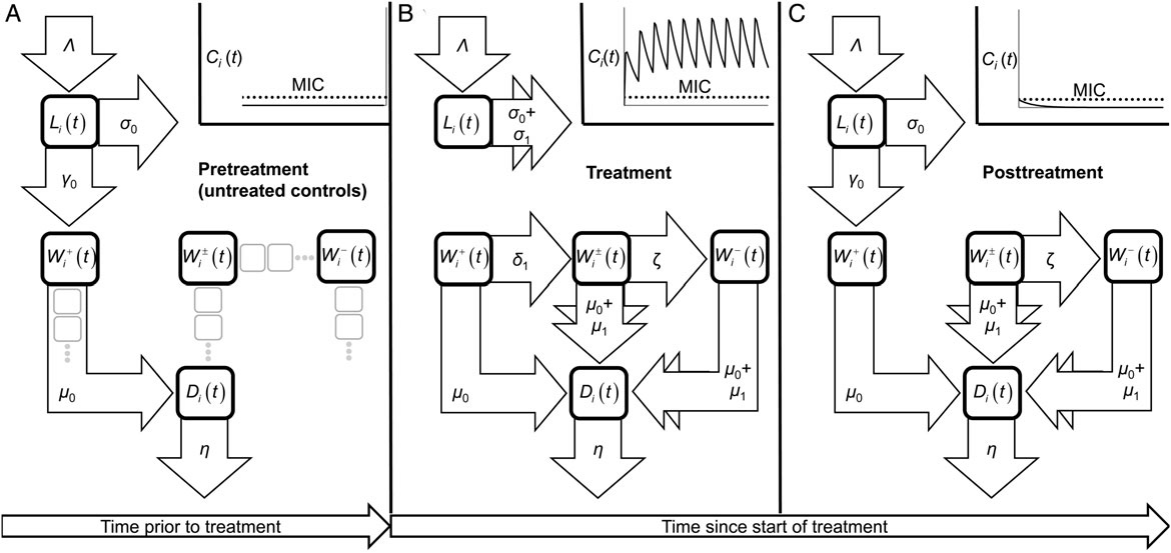
Schematic representation of the model describing the dynamics of *Wolbachia* depletion from adult filariae coupled to the ensuing antifilarial effects. The variables *L_i_*(*t*), *W_i_*^+^(*t*), *W_i_*^±^ (*t*), *W_i_*^–^(*t*), and *D_i_*(*t*) denote, respectively, the mean number of infective (L3) larvae, and *Wolbachia-*positive, *Wolbachia-*depleted, *Wolbachia-*negative, and adult dead worms in trial participants given drug regimen *i.* The smaller gray squares in (*A*) indicate the positions of latent compartments used to assess model structural uncertainty on parameter estimates. These latent compartments are omitted from (*B*) and (*C*) for clarity. Temporal dependence in certain per worm rate parameters [eg, *δ_i_*(*t*)] is governed by whether the average blood plasma concentration of doxycycline in participants given drug regimen *i*, *C_i_*(*t*), is greater than the minimum inhibitory concentration (MIC) against *Wolbachia* (Supplementary Methods, Pharmacokinetic Model). Infective, L3 larvae are acquired at rate *Λ*, die at (per capita) rate *σ_i_*(*t*), and progress toward *Wolbachia-*positive adult worms at rate *γ_i_*(*t*). When the concentration of doxycycline is above the MIC, *σ_i_*(*t*) = *σ*_0_ + *σ*_1_ and *γ_i_*(*t*) = 0 (*B*). Below the MIC, *σ_i_*(*t*) = *σ*_0_ and *γ_i_*(*t*) = *γ*_0_. *Wolbachia-*positive worms die at rate *µ*_0_, becoming immunohistologically or morphologically identifiable as dead, before being resorbed at rate *η*. Depletion of *Wolbachia* occurs at rate *δ_i_*(*t*) = *δ*_1_ while the concentration of doxycycline is greater than the MIC; no depletion occurs otherwise [*δ_i_*(*t*) = 0]. *Wolbachia-*depleted worms become immunohistologically observable as *Wolbachia* negative at rate *ζ*. Both *Wolbachia-*depleted and *Wolbachia-*negative worms incur a higher mortality rate than their *Wolbachia-*positive counterparts, *µ*_0_ + *µ*_1_ (the combination of background mortality and doxycycline-induced, excess mortality).

Numbers of larval and live and dead adult *O. volvulus* are modeled by a system of ordinary differential equations, represented schematically in Figure 1. Participants are infected with L3 larvae at a constant rate, the force of infection (the proportion of trial participants relative to the whole population is too small to affect the intensity of transmission and the impact of possible seasonal fluctuations in fly biting is negligible; Supplementary Analyses, Seasonality in the Force of Infection). The larval state is unobserved in the data but captures the approximate 2-year prepatent period of *O. volvulus* [12]. Live adults are divided into *Wolbachia*-positive, *Wolbachia*-depleted, and *Wolbachia*-negative states. The rate of progression between *Wolbachia*-positive and *Wolbachia*-depleted states is governed by the modeled concentration of doxycycline. *Wolbachia-*depleted worms pass into the *Wolbachia-*negative state at a constant rate (independent of the concentration of doxycycline), where they become immunohistologically detectable as *Wolbachia-*negative. The *Wolbachia*-depleted state is unobserved, but its inclusion permits the protracted decline in *Wolbachia-*positive worms while allowing for the possibility that some treatment regimens were suboptimal and precipitated systematically lower numbers of *Wolbachia-*negative worms. Macrofilaricidal activity is embodied by an excess mortality of *Wolbachia*-depleted and *Wolbachia*-negative worms such that their expected life span can be shorter than the 8 to 12 years [13] of worms with a full complement of *Wolbachia*. Prophylactic activity of doxycycline is modeled by an excess mortality of immature worms and inhibition of their progression to adults, ensuring that larvae cannot establish as adults during treatment, consistent with experimental observations on the effects of tetracyclines on filarial parasites of rodents [14, 15].

### Doxycycline Pharmacokinetics and Pharmacodynamics

A pharmacokinetic (PK) and pharmacodynamic (PD) submodel relates doxycycline dose and concentration to ensuing anti-*Wolbachia* and antifilarial effects (Supplementary Methods, Pharmacokinetics Model and Pharmacodynamics Model). Participants' blood plasma concentrations of doxycycline are modeled using a 2-compartment PK model with treatment duration 4, 5, or 6 weeks, dose of 100 or 200 mg daily, and published PK/PD parameter estimates (Supplementary Table 2). Doxycycline is assumed to exhibit binary concentration-dependent activity or inactivity, depending on whether the concentration is above or below the minimum inhibitory concentration, respectively (Supplementary Methods, Pharmacodynamics Model), reflecting the primarily bacteriostatic action of tetracycline antibiotics [16].

### Statistical Model

The dynamic models define means in groups of individuals taking a particular drug regimen. However, the available data are individual-based and longitudinal and, consequently, observations from the same individual at different times (repeated measures) are not independent. Dependence arises from variation unaccounted for by the measured covariates, including nonspecific PK/PD or parasite population dynamic variation. The mean-based dynamic model is therefore augmented with individual-specific random effects, permitting the modeled proportion of live worms and (live) *Wolbachia*-positive worms to vary among participants (Supplementary Methods, Statistical Model). This accounts for correlation among repeated measures and suitably inflates estimates of parameter uncertainty. State probabilities, rather than absolute numbers, are modeled to “normalize” the data, nullifying the effects of variation in the force of infection among participants.

### Parameter Inference

Inference is conducted in a Bayesian framework [17], integrating parameter uncertainties into estimated parameter posterior distributions (posteriors) using bespoke Markov chain Monte Carlo techniques [18, 19] (Supplementary Methods, Parameter Inference). Parameters with prior information available in the literature (9 in total) are assigned informative uniform prior distributions (priors) with bounds defined by the range of published estimates (Supplementary Table 2). Parameters without prior information (7 in total as listed in Table 2, excepting *µ*_0_) are assigned uninformative priors (Supplementary Table 2 and Supplementary Figure 1). The adequacy of the model fit to the data is confirmed by inspection of standardized residuals (Supplementary Analyses, Diagnostic Checks).

**Table 2.** Parameter Posteriors Estimated by Fitting the Dynamic Model to Doxycycline Clinical Trial Data on the *Wolbachia* Status and Vitality of Female *Onchocerca volvulus* From 182 Participants

Parameter	Definition	Posterior Mean^a^ (95% BCI)	Range of Posterior Mean^b^	Units
1/(*µ*_0_ + *µ*_1_)	Life expectancy of *Wolbachia-*depleted adult worms	2.1 (1.7–2.7)	2.1–3.0	Years
1/*ζ*	Average clearance time of depleted *Wolbachia* populations from adult worms	70 (56–85)	70–87	Days
1/*η*	Average resorption time of dead adult worms	1.9 (1.4–2.5)	1.8–1.9	Years
*π*	Probability that a worm is correctly identified as *Wolbachia* positive	0.97 (0.95–0.99)	0.97–0.97	None
*υ*_1_	Inverse variance (precision) among individual hosts in the proportion of live female worms	5.1 (3.0–8.7)	5.1–5.6	None
*υ*_2_	Inverse variance (precision) among individual hosts in the proportion of *Wolbachia-*positive female worms	4.2 (1.8–9.5)	2.9–4.8	None

Abbreviation: BCI, Bayesian credible interval.

^a^ Reported to 2 significant figures.

^b^ Range of posterior means calculated using different model structural configurations.

### Model Structural Uncertainty

The “one-state, one-compartment” structural configuration implicitly assumes an exponential distribution of transition times between contiguous states. This assumption can affect parameter estimates [20] and is explored by reconfiguring the model into a “one-state, multiple-compartment” structure using “latent” compartments (Figure 1). Details are given in Supplementary Analyses, Model Structural Uncertainty.

## RESULTS

### Dynamics of *Wolbachia* Depletion

**Figure 2.**
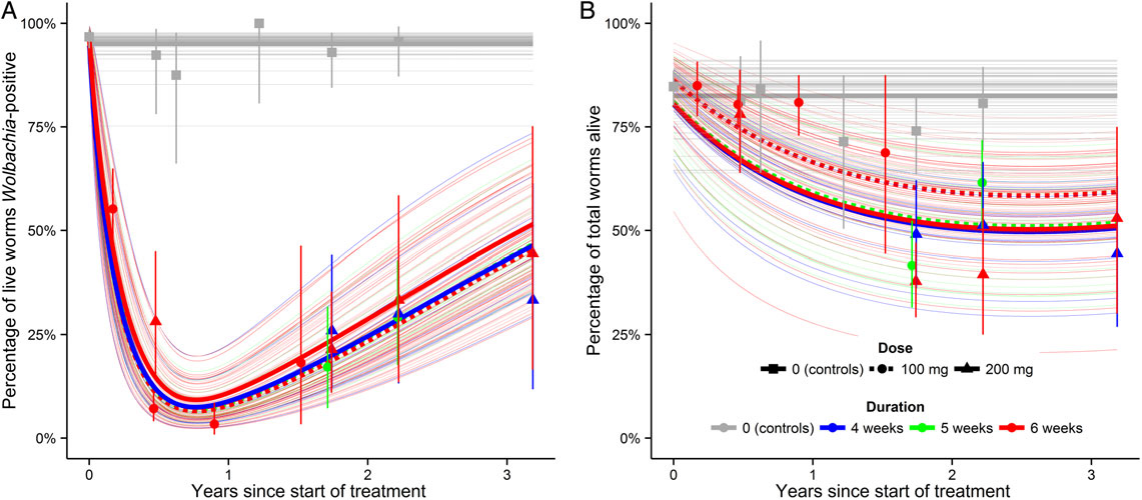
Fitted and observed proportions of female *Onchocerca volvulus* by *Wolbachia* status and viability, against time since the start of doxycycline treatment. The proportion of (live) *Wolbachia-*positive female worms is depicted in (*A*) and the proportion of (total) live female worms is depicted in (*B*). In each panel, the thick blue, green, and red lines denote, respectively, the marginal percentage of worms (ie, averaged over trial participants) receiving doxycycline for 4, 5, or 6 weeks respectively. Solid and broken lines indicate doses of 100 mg and 200 mg, respectively. The thick solid gray line denotes the marginal percentage of worms in placebo-treated and untreated control patients (Table 1). Thin lines, applying the same color scheme to indicate different treatment regimens, represent (posterior mean) individual patient trajectories. Variation among individual trajectories in the percentage of (total) live worms and the percentage of (live) *Wolbachia-*positive worms is governed by the precision parameters *υ*_1_ and *υ*_2_ (Table 2), respectively. Data points represent the observed data grouped (for presentation purposes only; the model is fitted to the individual patient data) by follow-up times (follow-up times disaggregated by month of follow-up are given in Supplementary Table 1) and plotted at the median follow-up time per group. The color of the data points corresponds to the duration of treatment in the same manner as the model-predicted proportions. A treatment dose of 100 mg or 200 mg per day is indicated by a circle or a triangle, respectively; data from untreated or placebo treated patients are indicated by (gray) squares. Vertical bars represent exact (likelihood profile) 95% confidence intervals for the observed data. Note that data from different regimens collected at proximate times are very similar, verifying that different treatment regimens are of approximately equivalent efficacies (Figure 3). The progressive increase in the proportion of live, *Wolbachia*-positive worms after 9.5 months is not due to recrudescence of the bacteria, but to both acquisition of new, drug-naive worms with a full complement of bacteria, and increased mortality of treated worms.

The observed and estimated percentages of *Wolbachia*-positive *O. volvulus* with time after the start of doxycycline treatment or matching placebo are illustrated in Figure 2*A*. Parameter posterior estimates are summarized in Table 2. From the *Wolbachia-*depleted state, it takes, on average, 70–87 days before the *Wolbachia* populations are cleared and worms are detected as *Wolbachia* negative. This drives the protracted decline in the percentage of *Wolbachia*-positive worms, which has a nadir approximately 9.5 months after the start of doxycycline treatment.

The rate of depletion from the *Wolbachia-*positive to the *Wolbachia-*depleted state is unidentifiable (Supplementary Figure 1), suggesting that treatment regimens are equally effective in depleting *Wolbachi*a; none of the regimens are either too short, or given at insufficient dose, to elicit systematically lower percentages of *Wolbachia-*positive worms.

The dynamics of *Wolbachia* depletion followed by clearance indicate that (1) the time taken for *Wolbachia* populations to become eventually extinct is much longer than the time taken for doxycycline to push the bacteria populations into terminal decline, and/or (2) it takes a long time for dead *Wolbachia* or wolbachial remnants to be cleared by the worm and appear negative by immunohistology.

### Dynamics of Reinfection

The increasing proportion of *Wolbachia-*positive worms after 9.5 months is driven partly by reinfection with doxycycline-naive, *Wolbachia-*positive worms, and partly by the increased mortality of *Wolbachia-*depleted and *Wolbachia-*negative worms. These effects dilute the directly observable effect of doxycycline treatment on wolbachial loads to a degree that depends on when observations are made after treatment (Figure 2*A*).

### Therapeutic Efficacy of Doxycycline

**Figure 3.**
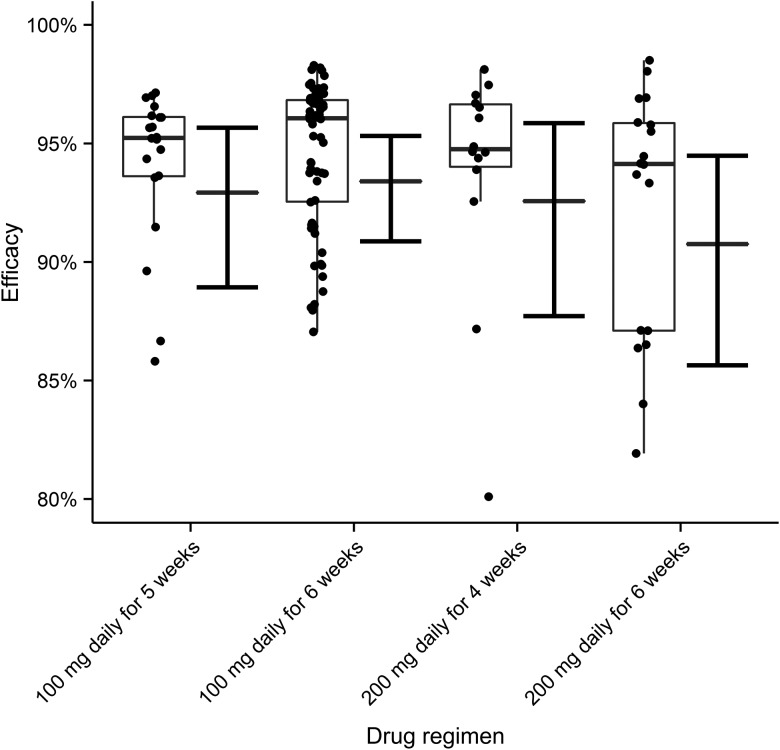
The efficacy of doxycycline in depleting *Wolbachia* from adult female *Onchocerca volvulus* estimated for different drug regimens. Efficacy is defined as the maximum proportional reduction in the percentage of adult female *O. volvulus* positive for *Wolbachia*. The circles represent posterior medians of the estimates for each of the 114 treated trial participants. These are overlaid with a box-and-whisker plot depicting the interquartile range (IQR) of individual estimates (box) and extending to 1.5 × IQR (whiskers). The solid horizontal bar within the box depicts the median of the individual efficacy estimates. The solid horizontal bars beside the individual estimates represent the marginal efficacy averaged over all patients receiving a particular treatment regimen. The accompanying error bars represent 95% Bayesian credible intervals (BCIs). The values and BCIs of the marginal efficacies are as follows: 100 mg daily for 5 weeks, 93% (89%–96%); 100 mg daily for 6 weeks, 93% (91%–95%); 200 mg daily for 4 weeks, 93% (88%–96%); 200 mg daily for 6 weeks, 91% (86%–94%).

**Figure 4.**
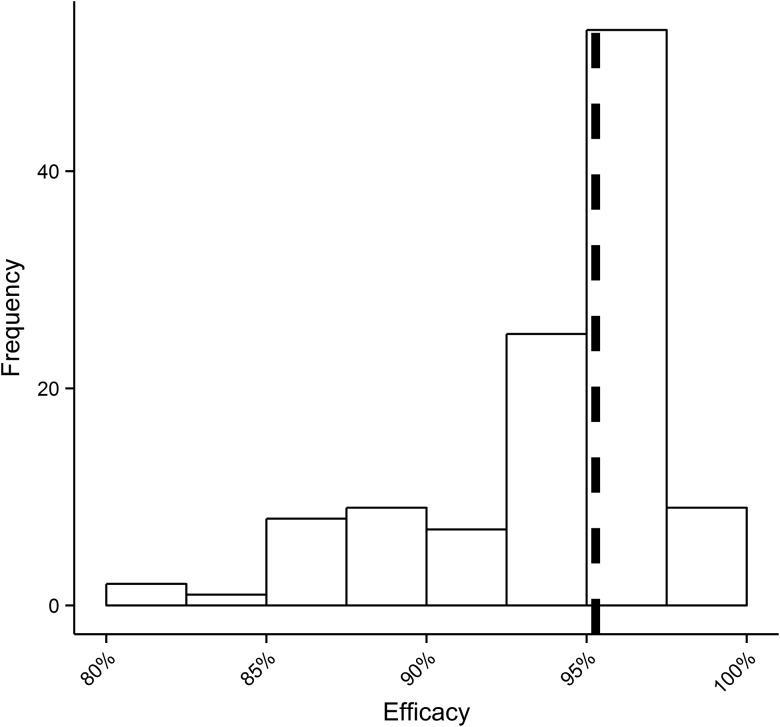
The distribution of efficacies estimated for individual patients aggregated by treatment regimen. Efficacy is defined as the maximum proportional reduction in the percentage of adult female *Onchocerca volvulus* positive for *Wolbachia*. Efficacy estimates represent posterior medians for each of the 114 treated patients. The vertical dashed line is the median of the individual estimates indicating that the efficacy of doxycycline is > 95% in the majority of patients.

The therapeutic efficacy of doxycycline is defined as the maximum proportional reduction in the percentage of adult female *O. volvulus* positive for *Wolbachia*. This is not directly observable because (1) data are not available from all participants, at times when the minimum percentage of *Wolbachia-*positive worms would be observed; (2) the pretreatment percentage of *Wolbachia*-positive worms for each participant is estimated from the model; and (3) some (approximately 3%) *Wolbachia*-positive worms are missed by the estimated 97% sensitivity (Table 2) of *Wolbachia* detection methods. The efficacy estimates depicted in Figures 3 and 4 indicate that efficacy is >91% on average, >95% in the majority of participants, and that there is no statistically significant difference among treatment regimens. The model's structural configuration has a negligible effect on efficacy estimates (Supplementary Figure 4).

### Macrofilaricidal Activity

The estimated 2- to 3-year life expectancy of female worms depleted of *Wolbachia* (Table 2) is 20%–30% of the average 8- to 12-year life expectancy [12] of *Wolbachia-*positive worms. This reduced longevity drives the prolonged decline in the percentage of live *O. volvulus*, which troughs at approximately 50%, 2.5 years after the start of treatment (Figure 2*B*). Despite the fact that the directly observable maximum decline in the percentage of live worms is just 30%, the reduction in longevity of adult worms is considerable, illustrating that the true macrofilaricidal activity of doxycycline is greater than can be detected using direct methods of evaluation.

## DISCUSSION

Quantification of the antifilarial effects of anti-*Wolbachia* therapy has hitherto been restricted to outcomes, such as the percentage of *Wolbachia-*positive, negative, or dead *O. volvulus*, measured at various times after treatment in clinical trials. The dynamics of these outcomes, driven by post-treatment reinfection and the slow turnover of long-lived filarial populations, precludes the use of traditional meta-analytical approaches to assess antifilarial activity. The model presented here overcomes these problems by coupling the dynamics of doxycycline-induced *Wolbachia* depletion with the ensuing antifilarial effects. By fitting the model to clinical trial data on the *Wolbachia* and viability status of female *O. volvulus*, we estimate that (1) the efficacy of doxycycline (the maximum proportional reduction in the percentage of adult female *O. volvulus* positive for *Wolbachia*) therapy is >91% on average and >95% in the majority of participants, irrespective of treatment regimen, and (2) the macrofilaricidal effect of eliminating *Wolbachia* is a 70%–80% reduction in parasite life span.

Accounting for the competing effects of chemotherapy and post-treatment reinfection by drug-naive pathogens on directly observable measures of therapeutic efficacy is generally applicable to clinical trials conducted in endemic settings. Rarely, attempts have been made to restrict reinfection. For example, household insecticidal control of triatomine vectors of *Trypanosoma cruzi* was used to reduce reinfection during a trial of benznidazole for Chagas disease [21]. More commonly, exogenous reinfections are distinguished from endogenous infections (representative of treatment failure) at the evaluation stage. For example, molecular (genetic) analytical approaches are used to identify post-treatment reinfections with the etiological agents of duodenal ulcers [22], tuberculosis [23], malaria [24], and intestinal schistosomiasis [25]. Morphological characteristics have been used to identify young, newly acquired *O. volvulus*, permitting adjustment of the observed proportion of dead worms by the number estimated to have been acquired aftertreatment and prior to evaluation [7]. However, this approach does not address the further complication—particularly relevant to the protracted population dynamics of filarial parasites—that observed outcomes are dependent not only on treatment efficacy but *when* after treatment observations are made. Consequently, to permit simultaneous analysis of data collected at different follow-up times and to account for the vagaries of the epidemiological context, fitting a population dynamics model able to capture the effects of reinfection on outcome measures provides a powerful solution [26, 27].

The clinical trials from which the data are derived aimed to demonstrate a statistically significant difference between outcomes in treated vs control participants. The modeling shows that follow-up times with maximum statistical power to achieve this aim depend on the outcome in question. For example, live *Wolbachia*-positive *O. volvulus* (detectable by immunohistology) are least abundant 9.5 months after the start of treatment, whereas the corresponding figure for dead worms is 2.5 years (100% percentage of live worms in Figure 2). The clinical trials so far undertaken have included follow-up times that fall broadly within these optimal time frames, depending on whether the focus was on the doxycycline-induced depletion of *Wolbachia* [9] or on the killing of adult worms [8, 10]. Data have also been collected at less than optimal follow-up times. Here this has proved serendipitous to estimating our model parameters to an acceptable accuracy, highlighting that to parameterize mathematical models, a broad range of follow-up times is essential.

The 93% average efficacy for patients receiving the 4-week course of doxycycline (Figure 3), the shortest that has hitherto been trialed (although a trial including a 3-week course of doxycycline has recently been completed but not yet evaluated; see http://isrctn.org/ISRCTN06010453), is impressive and above the 90% empirical threshold for optimum antifilarial effects [2]. The absence of a statically significant difference among treatment regimens suggests that the 4-week course, as previously proposed [28], is sufficient for anti-*Wolbachia* therapy. Little substantive insight into the minimum therapeutically sufficient duration is possible here, as none of the regimens precipitate systematically lower percentages of *Wolbachia-*positive worms in treated participants. However, evidence from a small number of individuals who dropped out of treatment after 2 weeks suggests that this duration is insufficient. Therefore, it is reasonable to conclude that the minimum therapeutically sufficient course of doxycycline alone is between 2 and 4 weeks. Ongoing work by the Anti-*Wolbachia* Consortium (A•WOL, http://www.a-wol.net/) has shown in animal models and clinical trials that anti-*Wolbachia* drug combinations can markedly reduce treatment duration [29].

Doxycycline is currently the best option for treatment of onchocerciasis or lymphatic filariasis in patients attending clinical settings [6]. Doxycycline is the only available safe and effective curative therapy for onchocerciasis. For lymphatic filariasis, doxycycline does not have the side effects associated with the rapid microfilaricidal activity of diethylcarbamazine and ameliorates morbidity [2, 30]. Doxycycline has been considered incompatible with treatment in community settings because of perceived issues of compliance with the treatment course, contraindications in pregnant/breastfeeding women and children aged <8 years, and inadequate cost–benefit ratios [2, 6]. However, results from a trialed community-directed treatment approach suggested that high levels of coverage and compliance with 6 weeks of doxycycline could be achieved [31], with long-term reductions in microfilaridermia that are sustained for at least 4 years after delivery [32]. Moreover, A•WOL aims to find new anti*-Wolbachia* treatment regimens, including novel antimicrobial agents and alternative combinations of existing antimicrobials that are efficacious over a shorter duration and may be safe for currently excluded groups. It is thus envisaged that anti-*Wolbachia* therapy will in the future have a wider scope of application [29].

There are 3 main scenarios in which anti-*Wolbachia* therapy could be administered to populations on a “test and treat” basis. First, the delivery of community-directed treatment with ivermectin is impeded in areas where loiasis is coendemic with onchocerciasis and/or lymphatic filariasis because of the risk of severe adverse events [33]. This may hinder progress toward the World Health Organization's 2020 control and elimination goals [34]. Anti-*Wolbachia* therapy offers a viable alternative in such areas because *Loa loa* (the causal agent of loiasis) is one of the few filarial species without *Wolbachia* and so it is unaffected by treatment [1, 2]. Second, in those foci where elimination is deemed achievable, and transmission has been suppressed but not yet interrupted, targeted anti-*Wolbachia* therapy may be used to “mop up” residual infections [35, 36]. Third, anti-*Wolbachia* therapy would provide an invaluable backup approach in regions where suboptimal responses to ivermectin have been reported [37, 38].

Existing filariasis transmission models need to capture the effects of anti-*Wolbachia* therapy on filarial population dynamics to guide and assess the effectiveness and cost-effectiveness of new anti-*Wolbachia* intervention strategies. The presented model is designed to be generalizable to any anti-*Wolbachia* therapy of a human filariasis. Once linked with transmission dynamics models, it will inform the use of anti-*Wolbachia* therapy as an alternative and/or complementary strategy to help the ongoing onchocerciasis and lymphatic filariasis control and elimination programs, particularly in epidemiological settings where current strategies are deemed insufficient to achieve the 2020 goals [34].

## Supplementary Data

Supplementary materials are available at *Clinical Infectious Diseases* online (http://cid.oxfordjournals.org). Supplementary materials consist of data provided by the author that are published to benefit the reader. The posted materials are not copyedited. The contents of all supplementary data are the sole responsibility of the authors. Questions or messages regarding errors should be addressed to the author.

Supplementary Data

Supplementary Data
