# Supplementary material for: Therapeutic Efficacy and Macrofilaricidal Activity of Doxycycline for the Treatment of River Blindness
Source: Clin Infect Dis. 2014 Dec 23;60(8):1199–207. doi: 10.1093/cid/ciu1152 (PMC4370165; doi:10.1093/cid/ciu1152)
Supplement: Supplementary Data [file supp_60_8_1199__index.html]

Therapeutic Efficacy and Macrofilaricidal Activity of Doxycycline for the Treatment of River Blindness — Therapeutic Efficacy and Macrofilaricidal Activity of Doxycycline for the Treatment of River Blindness — Supplementary Data 

# Therapeutic Efficacy and Macrofilaricidal Activity of Doxycycline for the Treatment of River Blindness

## Supplementary Data

Supplementary Data

**Files in this Data Supplement:**

- Supplementary Data - Docx file
