# Supplementary material for: Therapeutic Efficacy and Macrofilaricidal Activity of Doxycycline for the Treatment of River Blindness
Source: Clin Infect Dis. 2014 Dec 23;60(8):1199–207. doi: 10.1093/cid/ciu1152 (PMC4370165; doi:10.1093/cid/ciu1152)
Supplement: Supplementary Data [file supp_ciu1152_ciu1152supp.docx]

# Supplementary Material

The therapeutic efficacy and macrofilaricidal activity of doxycycline for the treatment of river blindness

Martin Walker, Sabine Specht, Thomas S. Churcher, Achim Hoerauf, Mark J. Taylor and María-Gloria Basáñez

# Supplementary Methods

## Systematic review

We searched for clinical trials on doxycycline for the treatment of onchocerciasis on PubMed using the terms “onchocerciasis”, “*Wolbachia*”, “doxycycline” and “trial”. The search identified 11 publications. Four were on the trials of Hoerauf *et al* [1-4] which provided the data analysed in this article; one was a reanalysis of some of these data to identify morphologically *Onchocerca volvulus* acquired prior to post-treatment follow-up [5]. Two articles pertained to trials of antibacterials other than doxycycline and two were review articles. The trial described by Tamarozzi *et al* [6] on the long-term (four-year) effectiveness of community-wide doxycycline treatment [7], did not collect data on adult worms. The trial described by Turner *et al.* [8], conducted in a loiasis-onchocerciasis co-endemic setting, measured similar outcomes as Hoerauf *et al*. albeit with *Wolbachia* loads of individual *O. volvulus* measured by quantitative PCR rather than immunohistology. Consequently, these data were not suitable for the analysis presented here. The complete dataset analysed in this paper and disaggregated by month of follow-up is summarised in Supplementary Table 1.

## Participant compliance and follow-up

Combining all individual participant data (Table 1; Supplementary Table 1), 129 of 140 enrolled participants completed their course of doxycycline, corresponding to an overall compliance rate of 92%; non-compliant participants were not followed up. Of the 129 compliant participants, 114 (88%) were nodulectomized at least once [1, 2, 3]. Data on the *Wolbachia* status and vitality of female adult *O. volvulus* from these 114 participants are analysed in the current work (Table 1; Supplementary Table 1) to determine the anti-*Wolbachia* and antifilarial properties of doxycycline therapy.

It is unlikely that data missing from the 8% of non-compliant participants and the 12% of individuals absent for nodulectomy led to any appreciable bias in the results. First, in total, 114 out of 140 (81%) enrolled participants were successfully followed up. Hence, any systematic difference in the anti-*Wolbachia* and consequent antifilarial response to doxycycline in the missing 19% would have had to be extremely pronounced to have had a substantive influence on the final results. Second, an association between drug response and compliance can arise when individuals start ‘feeling better’ as a result of treatment and are therefore more motivated to continue (although the converse is also conceivable; that ‘feeling better’ decreases one’s motivation to continue). Hence, compliance may be higher among individuals in whom treatment is more (or less) efficacious. However, for anti-*Wolbachia* onchocerciasis therapy this mechanism does not seem particularly plausible since the protracted decline in microfilariae—which cause onchocercal pathology—induced by doxycycline therapy means that alleviation of associated disease sequelae will occur after cessation of treatment. Third, the majority of individuals completing treatment but absent for nodulectomy had left the local community; it seems unlikely that these individuals had a profoundly different anti-*Wolbachia* and antifilarial response to doxycycline compared to individuals who remained.

| **Supplementary Table 1. Summary of nodulectomy data collated from three clinical trials on the effects of doxycycline on female *Onchocerca volvulus*, disaggregated by month of follow-up** | | | | | | | | | |
| --- | --- | --- | --- | --- | --- | --- | --- | --- | --- |
| Regimen | | Participants completing treatment | Follow-up time after treatment | | Participants followed-up | Extirpated nodules | Female worms by *Wolbachia* status | | Dead female worms |
| Duration (wks^a^) | Dose (mg^b^ day^-1^) |  | Months | Days |  |  | Positive | Negative |  |
| 4 | 200 | 14 | 20 | 630-638 | 7 | 36 | 7 | 20 | 28 |
|  |  |  | 27 | 810-817 | 7 | 23 | 6 | 14 | 19 |
|  |  |  | 39^c^ | 1162-1163 | 2 | 13 | 4 | 8 | 15 |
| 5 | 100 | 20 | 20 | 624-630 | 13 | 52 | 6 | 29 | 49 |
|  |  |  | 27 | 804-812 | 12 | 44 | 14 | 34 | 30 |
| 6 | 100 | 62 | 2 | 56-64 | 19 | 51 | 53 | 43 | 17 |
|  |  |  | 5.5 | 134-175 | 41 | 112 | 14 | 183 | 48 |
|  |  |  | 11 | 309-329 | 13 | 45 | 3 | 86 | 21 |
|  |  |  | 18 | 552-557 | 4 | 9 | 2 | 9 | 5 |
| 6 | 200 | 18 | 6 | 172-181 | 11 | 19 | 9 | 23 | 9 |
|  |  |  | 20 | 634-638 | 13 | 62 | 9 | 33 | 69 |
|  |  |  | 27 | 810-817 | 5 | 27 | 5 | 10 | 23 |
|  |  |  | 39^c^ | 1162 | 2 | 7 | 4 | 5 | 8 |
| 6 | placebo | 23 | 5.5 | 172-176 | 7 | 7 | 17 | 0 | 1 |
|  |  |  | 6 | 180-183 | 6 | 7 | 9 | 3 | 5 |
|  |  |  | 9 | 273 | 1 | 9 | 12 | 1 | 3 |
|  |  |  | 14 | 445-446 | 2 | 9 | 15 | 0 | 6 |
|  |  |  | 20 | 635-639 | 15 | 42 | 53 | 4 | 20 |
|  |  |  | 27 | 810-816 | 5 | 29 | 44 | 2 | 11 |
| Untreated |  | 45 | NA^d^ | NA | 45 | 142 | 236 | 8 | 44 |

^a^weeks; ^b^milligrams; ^c^data from patients nodulectomizd at the 39 month follow-up time were not presented as part of the main analysis in the original study [8]; ^d^not applicable

## Pharmacokinetic model

A simple two-compartment pharmacokinetic (PK) model [9-11] based on ordinary differential equations (ODEs) was used to capture the dynamics in the amount, *A_i_*, of doxycycline in the body of a clinical trial participant taking drug regimen *i* at time *t* after the start of therapy,

$$\begin{matrix} \frac{dA_{ai}\left(t\right)}{dt}=u_{i}\left(\tau_{i}\right)-k_{a}A_{ai}\left(t\right) \\ \frac{dA_{i}\left(t\right)}{dt}=FA_{ai}\left(t\right)-kA_{i}\left(t\right). \end{matrix}$$

Here *A_ai_*  represents the amount of drug at the absorption site of the gut (predominantly the duodenum), *u_i_*(*τ_i_*) is the rate of influx of drug into the gut which can be 100 mg or 200 mg per day lasting for *τ_i_* = 4, 5 or 6 weeks, and *k_a_* is the rate of drug absorption from the gut. The variable *A_i_* denotes the amount of the drug in plasma, the bioavailability, *F*, indicates the fraction of the dose that is absorbed intact into the blood plasma, and *k_e_* is the rate of elimination of the drug (predominantly via the kidneys). The rate of elimination is frequently characterized by a drug’s half-life, *t*_1/2_, where

$$t_{1/2}=\frac{\ln2}{k_{e}}.$$

Generally, the effect of a drug on a pathogen is correlated with the free (unbound) plasma concentration of the drug rather than with the total amount within the body [11, 12]. The (apparent) volume of distribution, *V*, is a dilution space relating the total amount of drug within the body to the observed blood plasma concentration, *C_i_*(*t*),

$$C_{i}\left(t\right)=A_{i}\left(t\right)/V.$$

Pharmacokinetic parameter estimates were obtained from the published literature using data from studies on non-fasted adult volunteers who were given a standard doxycycline dose of either 100 mg or 200 mg, orally or intravenously (Supplementary Table 1).

## Pharmacodynamics model

Pharmacodynamics pertains to the dynamical response of an infectious agent to the presence of, or more specifically, the concentration of a drug [12]. Although blood plasma concentration is not identical to concentration within onchocercomas for adult worms, or within various tissues for migrating larvae [13], plasma concentration is frequently linked to drug action against infectious agents [12]. Besides, data are scarce on the tissue penetration of doxycycline, and non-existent on the penetration of doxycycline into onchocercomas, although the tissue distribution of doxycycline is relatively high [14] compared with other antimicrobials [15]. A frequently used pharmacodynamic (PD) model is the so-called *E*_max_ model [9, 11] which is a Hill equation [16] with different parameter interpretations,

$$E_{i}\left(t\right)=\frac{E_{\max}C_{i}^{H}\left(t\right)}{C_{50}^{H}+C_{i}^{H}\left(t\right)}.$$

Here *E_i_*(*t*) indicates the effect of a drug on a pathogen given a concentration *C_i_*(*t*) raised to the power *H* (the Hill coefficient), *E*_max_, the maximum effect of the drug, and *C*_50_, the concentration at which half the maximum effect is attained. For increasing values of *H*, the relationship between *E_i_*(*t*) and *C_i_*(*t*) (at a given time) becomes increasingly sigmoidal [9]. In this analysis, *E*_max_ was set equal to 1 such that *E_i_*(*t*) represents a proportion of the maximum attainable effect.

Doxycycline is primarily bacteriostatic [14]; above a minimum inhibitory concentration (MIC) it prevents bacterial reproduction. To simplify the *E*_max_ model [Equation ], it was assumed that doxycycline had no effect on *Wolbachia* at concentrations below its MIC (*E_i_*(*t*) *=* 0), and a maximum effect (*E_i_*(*t*) *= E*_max_ = 1) above its MIC. This behaviour is mimicked by giving *H* in Equation an arbitrarily large value (*H →* ∞) so as to render *C*_50_ equivalent to the MIC. The MIC of doxycycline against *Wolbachia pipientis—*the species of *Wolbachia* found in some arthropods and filarial nematodes [17]*—*is between 0.0625 and 0.125 mg/L [18, 19]. This is in line with the 0.06 – 0.25 mg/L range estimated for a wide variety of other rickettsial bacteria [20, 21].

It is noteworthy that the lack of PK data from trial participants precluded PK-PD sub-modelling at the level of the individual. In reality, it is well known that PK indicators, such as blood plasma concentration-time curves, vary considerably among individuals given the same dose of antimicrobial drug. Furthermore, the non-linear nature of the *E*_max_ model, relating drug concentration to *Wolbachia* depletion means that the amalgamation of individual patient heterogeneities into an average PK profile for a given drug regimen (dose, duration) does not necessarily correspond to the average of the individual anti-*Wolbachia* effects.

In general, while some PK variation tends to be explained by physiological differences among individuals, the residual variability in drug concentration tends to be pronounced enough that it is generally accepted that dose is a very poor measure of pathogen exposure [12]. That is, PK variability resulting from different doses given to patients matched by variables such as age, sex and general health status, tends to be overwhelmed by extraneous variation. Furthermore, any systematic differences in exposure resulting from dose, physiology, and / or differences in the exposure of different parasite life-stages, are unlikely to affect substantially the time during which exposure exceeds the MIC, which is the key determinant of efficacy in this model. This is because *Wolbachia* bacteria are extremely sensitive to tetracycline antibiotics (as indicated by the very low MIC), meaning that drug concentrations remain above the MIC for similar durations, irrespective of dose or a patient’s physiological characteristics. Consequently, the overriding determinant of the time that exposure exceeds the MIC is the duration of treatment, and because treatment was directly observed (see *Methods*, *Data*), this variable is homogenous among patients receiving the same drug regimen.

## Population dynamics model

The population dynamics model is mean-based and deterministic and comprises a series of ODEs. Model equations are presented separately for the per host mean number of female *Onchocerca volvulus* in different parasite stages and states; namely, infective larvae inoculated into the host via simuliid vectors, *Wolbachia-*positive adult worms, *Wolbachia-*depleted adults, *Wolbachia-*negative adults and dead adult worms. Throughout the following mathematical definition, subscript *i* denotes the drug regimen taken by a trial participant (human host). The time after the start of anti-*Wolbachia* (doxycycline) therapy is denoted *t*.

The rate of change in the mean number of female incoming larvae is

$$\frac{dL_{i}\left(t\right)}{dt}=\Lambda-\left[\sigma_{i}\left(t\right)+\gamma_{i}\left(t\right)\right]L_{i}\left(t\right),$$

where Λ is the rate of infection with newly acquired larvae, the so-called force of infection (FOI). The FOI is a net influx rate, implicitly incorporating any density-dependent establishment effects [22, 23]. It is assumed to be constant among individuals since inter-participant heterogeneity does not affect the proportion of *O. volvulus* in each state. The time-dependent larval mortality rate is defined as

$$\sigma_{i}\left(t\right)=\sigma_{0}+\sigma_{1}E_{i}\left(t\right),$$

where *σ*_0_ denotes the background rate of larval mortality, *σ*_1_ is an excess mortality rate due to treatment, and *E_i_*(*t*) [Equation ] takes a value of 1 when the blood plasma concentration of doxycycline is greater than its MIC or 0 otherwise. Thus, larvae only incur and excess mortality rate while the concentration of doxycycline is greater than its MIC (i.e. when *E_i_*(*t*) = 1). The time-dependent larval development rate in Equation is defined as

$$\gamma_{i}\left(t\right)=\gamma_{0}\left[1-E_{i}\left(t\right)\right],$$

such that the rate of larval development (or progression towards adult worms) is equal to 0 (i.e. development is inhibited) while the concentration of doxycycline is greater than its MIC, or it is equal to *γ*_0_ (the background rate of development) otherwise.

The rate of change in the per host mean number of *Wolbachia* positive adult female worms is

$$\frac{dW_{i}^{+}\left(t\right)}{dt}=\gamma_{i}\left(t\right)L_{i}\left(t\right)-\left[\mu_{0}+\delta_{i}\left(t\right)\right]W_{i}^{+}\left(t\right),$$

where *µ*_0_ is the background mortality rate of *Wolbachia*-positive worms. The time-dependent rate of *Wolbachia* depletion is defined as

$$\delta_{i}\left(t\right)=\delta_{1}E_{i}\left(t\right),$$

such that *Wolbachia* are depleted at rate *δ*_1_ when the concentration of doxycycline is greater than its MIC or at rate 0 (i.e. no depletion) otherwise.

The rate of change in the per host mean number of *Wolbachia-*depleted female worms is

$$\frac{dW_{i}^{\pm}}{dt}=\delta_{i}\left(t\right)W_{i}^{+}\left(t\right)-\left(\mu_{0}+\mu_{1}+\zeta\right)W_{i}^{\pm}\left(t\right),$$

where *µ*_1_ is the excess mortality rate incurred by worms without *Wolbachia* and *ζ*  is the rate at which worms depleted of their *Wolbachia* populations become observable as *Wolbachia-*negative worms under immunohistological examination. Notice that even though the category of *Wolbachia*-depleted worms is modelled explicitly, in practice these worms are observed as *Wolbachia* positive in the data.

The rate of change in the per host mean number of *Wolbachia-*negative, live adult female worms is

$$\frac{dW_{i}^{-}\left(t\right)}{dt}=\zeta W_{i}^{\pm}\left(t\right)-\left(\mu_{0}+\mu_{1}\right)W_{i}^{-}\left(t\right).$$

The rate of change in the per host mean number of dead adult female worms is

$$\frac{dD_{i}\left(t\right)}{dt}=\mu_{0}W_{i}^{+}\left(t\right)+\left(\mu_{0}+\mu_{1}\right)\left[W_{i}^{\pm}\left(t\right)+W_{i}^{-}\left(t\right)\right]-\eta D_{i}\left(t\right),$$

where *η* is the rate at which dead worms are resorbed within nodules [24]. To complete the model definition, the total number of adult worms is

$$N_{i}\left(t\right)=A_{i}\left(t\right)+D_{i}\left(t\right),$$

where *A_i_*(*t*) is the total number of live worms, in all *Wolbachia* states.

## Statistical model

Let *j* indicate an individual and let *t_k_* indicate the time after the start of doxycycline treatment that an observation was made, making explicit that follow-up times were measured at discrete daily intervals and that data from different individuals were observed at multiple follow-up time points (i.e. *k ≥* 1) [25]. From the population dynamics model, the probabilities (Pr) that worms exposed to drug regimen *i* observed at *t_k_* are: a) alive, denoted *p_i_*(*t_k_*), and b) conditional on being alive, are *Wolbachia-*positive, denoted *q_i_*(*t_k_*), are given by

$\begin{matrix} \Pr\left[\mathrm{Alive}\right]=p_{i}\left(t_{k}\right)=\frac{A_{i}\left(t_{k}\right)}{N_{i}\left(t_{k}\right)}, \\ \Pr\left[Wolbachia \text{positive|Alive}\right]=q_{i}\left(t_{k}\right)=\frac{\pi\left[W_{i}^{+}\left(t_{k}\right)+W_{i}^{\pm}\left(t_{k}\right)\right]}{A_{i}\left(t_{k}\right)}, \end{matrix}$ (14)

where *W*^+^*_i_*(*t_k_*), *A_i_*(*t_k_*) and *N_i_*(*t_k_*) are discrete outputs from the population dynamics model. Parameter *π* is the probability (sensitivity) that a worm is correctly identified as *Wolbachia-*positive. This parameter was incorporated to capture empirically the small number of worms that were observed *Wolbachia-*negative in untreated control individuals.

We incorporated two random effects, denoted *b*_1_*_j_* and *b*_2_*_j_*, to render the probabilities *p_i_*(*t_k_*) and *q_i_*(*t_k_*) specific to individual *j*, defining a pair of generalized additive mixed models,

 (15)

Here, Φ is the cumulative distribution function of the standard normal distribution, also called the ‘probit’ link function, which facilitates efficient numerical parameter inference for binary regression models (see *Parameter inference*). The random effects *b*_1_*_j_* and *b*_2_*_j_* are assumed to be independently normally distributed among individuals on the (probit) link scale with precision (= 1 / variance) *υ*_1_ and *υ*_2_ respectively. This construction accounts for correlation among data by modelling the repeated measures (on the same individual at different points in time) as independent, *conditional on* the individual-specific random effects terms.

We derived a likelihood for the data by considering the joint probability of observing *y_ij_*(*t_k_*) live female worms and *z_ij_*(*t_k_*) *Wolbachia-*positive female worms, out of *n_ij_*(*t_k_*) females in total. This is given by the product Pr[*Y_ij_*(*t_k_*)] × Pr[*Z_ij_*(*t_k_*)|*Y_ij_*(*t_k_*)], analogous to the individual-based variant (i.e. after incorporation of the random effects terms *b*_1_*_j_* and *b*_2_*_j_*) of Pr[Alive] × Pr[*Wolbachia*positive | Alive] given in Equation (14). The likelihood of observations *y_ij_*(*t_k_*) and *z_ij_*(*t_k_*) (i.e. from individual *j* at time *t_k_*) is therefore given by the product of two binomial probabilities,

$L_{ijk}\left[y_{ij}\left(t_{k}\right),z_{ij}\left(t_{k}\right)|\mathbf{\theta}\right]=f\left[y_{ij}\left(t_{k}\right)|n_{ij}\left(t_{k}\right),p_{ij}\left(t_{k},\mathbf{\theta}\right)\right]\times f\left[z_{ij}\left(t_{k}\right)|\mathrm{y}_{ij}\left(t_{k}\right),\mathrm{q}_{ij}\left(t_{k},\mathbf{\theta}\right)\right],$ (16)

where *f*(k|n,p) is the probability mass function of the binomial distribution and **θ** represents the collection of model parameters hitherto defined and listed in Supplementary Table 2. The likelihood of the entre dataset (described by the vectors **y** and **z**) is given by the product of these individual-, time- and drug regimen specific likelihood contributions,$\mathrm{L}\left(\mathbf{y,z}|\mathbf{\theta}\right)=\prod_{i} \prod_{j} \prod_{k} L_{ijk}\left[y_{ij}\left(t_{k}\right),z_{ij}\left(t_{k}\right)|\mathbf{\theta}\right].$ (17)

## Parameter inference

Inference was conducted in a Bayesian framework using Markov chain Monte Carlo (MCMC) techniques [26]. Parameters with prior information in the published literature (9 in total) were assigned informative uniform prior distributions (priors) with bounds defined by the range of published estimates, while other parameters were assigned uninformative uniform priors (Supplementary Table 2).

Parameter (marginal) posterior distributions (posteriors) were sampled using Metropolis and Gibbs sampling techniques for, respectively, the dynamic model parameters and the random effects parameters (including precision terms *υ*_1_ and *υ*_2_). Specifically, population dynamics parameters were updated individually using a normal proposal distribution and accepting proposed moves using the Metropolis criterion (invoking the symmetry of the normal proposal distribution). The proposal distribution was dynamically updated during the burn-in phase using the empirical estimate of the posterior variance of the Markov chain [27, 28]. The dynamic model differential equations were solved for each proposed move to permit evaluation of the log-likelihood [the natural logarithm of Equation (17)]. This was achieved numerically, at discrete daily intervals (to match the observed data), using a variable step size numerical integration algorithm implemented by calling C code from R [29] using the deSolve package [30].

The posterior distributions of the random effects and precision parameters [*b*_1_*_j_*, *b*_2_*_j_*, *υ*_1_ and *υ*_2_ in Equation (15)] were sampled directly and in 4 blocks by Gibbs sampling, conditioning on the current values of all other parameters. Specifically, the random effects terms *b*_1_*_j_* and *b*_2_*_j_* were sampled using the latent variables approach for longitudinal binary probit models described by Albert, Chib and Carlin [31, 32] which exploits the conjugacy between the normally distributed random effects terms (*b*_1_*_j_* and *b*_2_*_j_*) and normally distributed latent variables that are simulated (conditional on the observed data) at each iteration of the Gibbs sampling algorithm (see Algorithm 4 in [32]). The posterior distributions of the precision parameters—describing in an inverse manner, the variability in the normally distributed random effects terms—were sampled by specifying uninformative conjugate gamma prior distributions with shape and scale parameters equal to 0.001 and 1000 respectively [26].

The Gibbs and adaptive-Metropolis sampling routines were written in C++ and interfaced with R using the Rcpp package [33]. Three starting values for the Markov chains were initialized in order to assess convergence on the parameter posteriors and to check that our conclusions were not sensitive to the choice of starting values [26]. The first 1000 iterations of each chain were discarded as burn-in and a further 4000 samples were used to estimate the marginal posterior distributions.

Posteriors distributions that were informed by the available data (i.e. substantively different from their prior distribution, Supplementary Figure 1) are summarized in Table 2, main text. The prior and estimated posterior distributions of all parameters are shown in Supplementary Figure 1. The therapeutic efficacy of doxycycline (the maximum proportional reduction in the percentage of *Wolbachia-*positive worms) was calculated by simulating, at daily intervals, trajectories from the dynamic model using parameters sampled from the (joint) parameter posterior distribution. Efficacy was expressed either as a marginal (population) quantity within strata defined by the different drug regimens (by averaging over variation among individual participants, see Figure 3, main text) or as a quantity specific to each individual participant (see Figure 3 and Figure 4, main text).

| Supplementary Table 2. Definitions of parameters and range of uniform prior distributions | | | | |
| --- | --- | --- | --- | --- |
| Symbol | Definition | Prior | Units | Ref. |
| *Population dynamics model* | | | | |
| *Λ* | Rate of infection of participant by L3 larvae; the ‘force of infection’ | arbitrary^a^ | year^-1^ |  |
| *γ*_0_ | *Per capita* rate of development from larval to adult worm | U(0.5, 1) | year^-1^ | [13, 48] |
| *σ*_0_ | Background larval mortality rate | U(0.25, 2)^b^ | year^-1^ | [13] |
| *σ*_1_ | Excess larval mortality rate induced by doxycycline | U(1, 365)^c^ | year^-1^ |  |
| *µ*_0_ | Background mortality rate of *Wolbachia*-positive adults | U(0.083, 0.125) | year^-1^ | [49] |
| *µ*_1_ | Excess mortality rate of worms depleted of *Wolbachia* | U(1.0×10^-9^, 100) | year^-1^ |  |
| *δ*_1_ | Rate of depletion of *Wolbachia* by doxycycline | U(1.0×10^-9^, 100) | day^-1^ |  |
| *ζ* | Rate of appearance of *Wolbachia*-depleted worms as *Wolbachia* negative | U(1.0×10^-9^, 100) | day^-1^ |  |
| *η* | Rate of resorption of dead worms | U(1.0×10^-9^, 100) | year^-1^ |  |
| *Pharmacokinetics-pharmacodynamics model parameters* | | | | |
| *k*_a_ | Rate of doxycycline absorption from the gut | U(7.6, 20)^d^ | day^-1^ | [50] |
| *k*_e_ | Rate of doxycycline elimination from blood plasma | U(0.67, 1.4)^d^ | day^-1^ | [14] |
| *V* | Volume of distribution | U(50, 80) | litres | [14] |
| *F* | Bioavailability | U(0.80, 0.99) |  | [14, 51] |
| MIC | Minimum inhibitory concentration of doxycycline against *Wolbachia* | U(0.0625, 0.125) | mg litre^-1^ | [18,19] |
| *Statistical model parameters* | | | | |
| *π* | Probability (sensitivity) that a worm is correctly identified as *Wolbachia*-positive | U(0, 1) | none |  |
| *υ*_1_ | Inverse variance (= precision) among individual hosts in the proportion of live female worms | G(0.001, 1000) | none |  |
| *υ_2_* | Inverse variance (= precision) among individual hosts in the proportion of *Wolbachia-*positive female worms | G(0.001, 1000) | none |  |

Distribution notation: U(*a*, *b*), uniform between *a* and *b*; G(*α*,*β*), gamma with shape *α* and scale *β*.

^a^ The force of infection has no effect on the model-predicted proportion of worms in each state under the assumption that it remains constant throughout the follow-up period; ^b^ motivated by the estimated 1 to 2 year estimated life-span of microfilariae with the lower and upper bounds doubled to reflect increased uncertainty on this parasite stage; ^c^ upper bound corresponds to an arbitrarily high excess mortality rate reflecting the circumstance that no pre-patent larvae exposed to doxycycline remain viable; ^d^ calculated from the range of published estimates on the corresponding half-life, i.e. the half-life of absorption ranges between 0.85 and 2.2 hours and the half-life of elimination ranges between 12 and 25 hours.


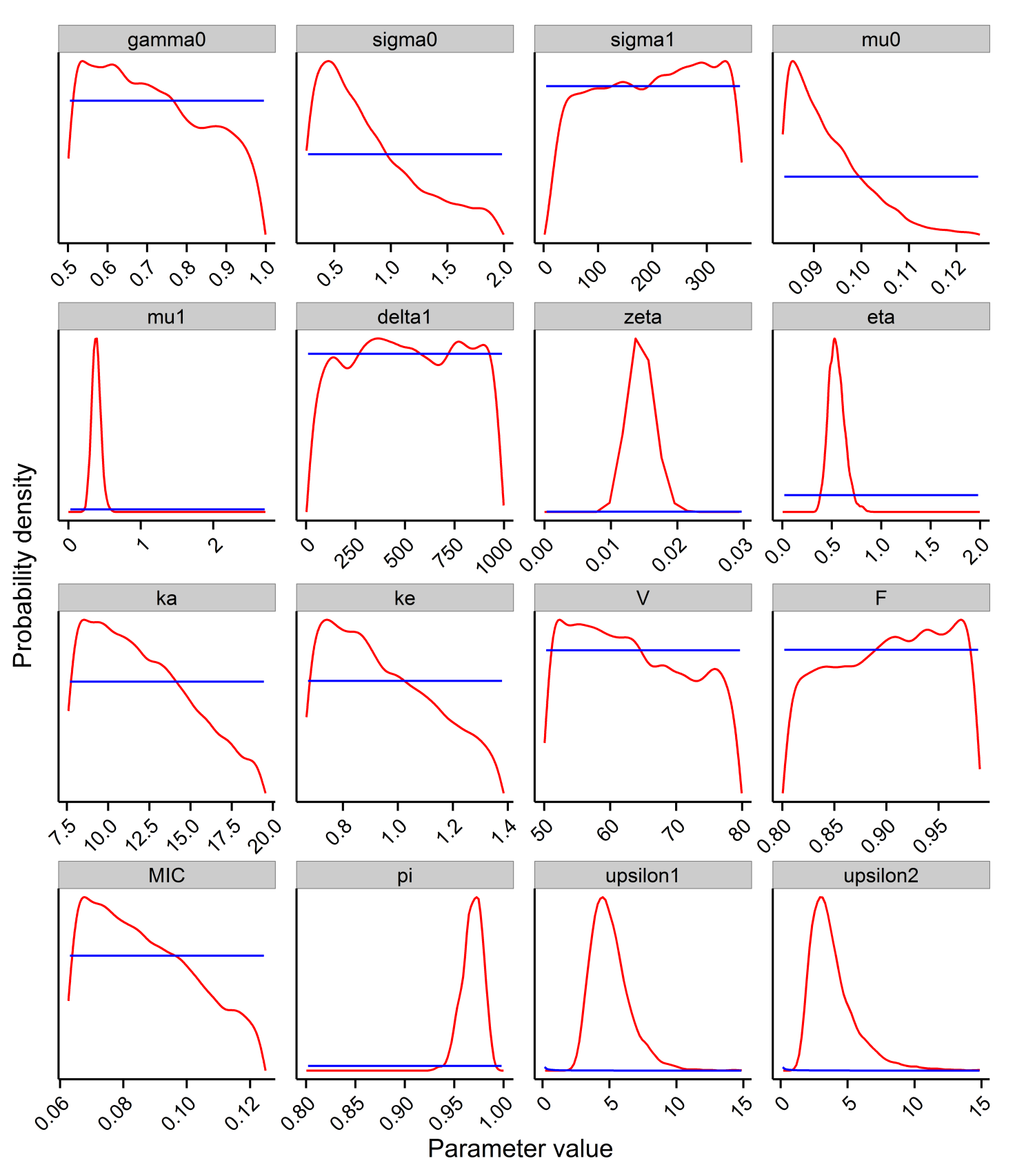


## Supplementary Figure 1. Prior (blue) and estimated posterior distributions (red) of the model parameters. Parameter definitions, prior parameterizations and units are listed in Supplementary Table 2. Parameters with a posterior substantively different from the prior are identifiable from the available data [e.g. the excess mortality rate of worms depleted of *Wolbachia*, *μ*_1_ (mu1)]. Note that the *x*-axis of identifiable parameters assigned uninformative priors (mu1, *μ*_1_; zeta, *ζ*; eta, *η*; pi, *π*; upsilon1, *υ*_1_, and upsilon2, *υ*_2_) are truncated so that the shape of the posterior is discernible.

# Supplementary Analyses

## Diagnostic checks

The adequacy of the model fit was assessed by inspecting the plot of the average standardized residuals against the average expected values of the response variables; namely, the number of live *O. volvulus* and the number of (live and) *Wolbachia-*positive female worms (Supplementary Figure 2). In a Bayesian context, residuals are stochastic; each set of **θ** from the posterior distribution yields a stochastic realization of the expected value of the response variable(s) (akin to the fitted values in a frequentist framework) and hence also a stochastic set of residuals. Consequently, diagnostic checks are typically undertaken using the average (arithmetic mean) of both the realized residuals and the expected values of the response variable(s) [26]. Standardization of the residuals was performed by dividing each raw realized residual by the realized value of the standard deviation of the response variable, which in binomial model framework is a function of the (realized) expected value.


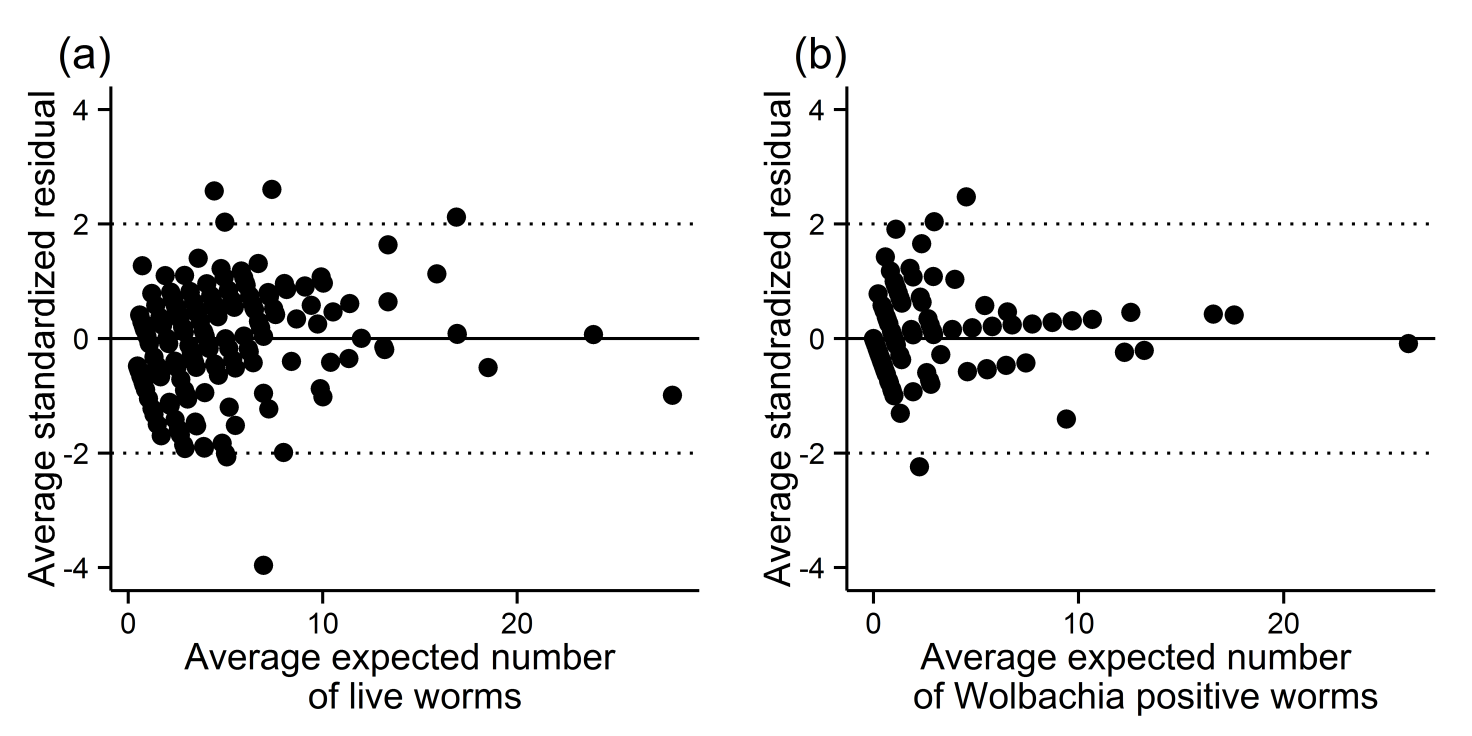


## Supplementary Figure 2. Bayesian residual analysis of the fitted model. Average standardized residuals are plotted against the average of the expected (fitted) number of live female *Onchocerca volvulus* (a) and the average expected (fitted) number of (live) *Wolbachia* positive female *O.* *volvulus* (b). The plots indicate that the model provides a satisfactory fit to the data; there is no ostensible systematic trend with the modelled expected values and the vast majority of values lie within 2 standard deviations of zero.

## Seasonality in the force of infection

The population dynamics model assumes that the rate at which an individual acquires new parasites (the force of infection, FOI) is constant over the follow-up period. However, a number of studies have shown that in West Africa—where all the original studies were conducted—the biting rate of the onchocerciasis blackfly vectors varies within the year, principally between wet and dry seasons [34, 35]. This likely induces, although not necessarily in a linear manner, seasonal variation in the FOI. We simulated fluctuations in the FOI to check that our model-based predictions of the proportion of live and *Wolbachia* positive female *O. volvulus—*which plainly form an integral component of the likelihood function within the statistical model (Equation 16)—were robust to this additional source of extraneous variation. We used a sinusoidal function that has been used to model seasonality in malaria transmission [36] to capture empirical fluctuations in the FOI. In particular, the function was parameterized to reflect qualitatively the pattern of seasonal blackfly biting reported in West Africa [37-39]. We considered two scenarios, both based on plausible but extreme seasonal patterns where transmission (a positive FOI) occurs only during a rainy season which lasts for four to five months. In the first, the peak FOI was assumed to correspond with the administration of doxycycline treatment, such that for the following four to five months the FOI rapidly declined. In the second, we assumed that doxycycline therapy was started at the nadir of the FOI such that for approximately four to five months after treatment, the FOI steadily increased. These scenarios are illustrated in Supplementary Figure 3a.

Trajectories from the dynamic model were simulated using parameters sampled from the (joint) parameter posterior distribution for a single ‘average’ patient (i.e. removing extraneous variation among individual hosts) experiencing a fluctuating FOI. Supplementary Figure 3b shows that the percentage of larval worms—which have a short life-span and higher population turnover rate compared to adult worms—is acutely affected by variation in the FOI. By contrast, the percentage of live adult worms *Wolbachia-*positive (Supplementary Figure 3c) and the percentage of the total number of adult worms that are alive (Supplementary Figure 3d) are both largely invariant to the pronounced (and probably somewhat exaggerated) oscillations in the FOI. This is because oscillations in the FOI occur on a time scale which is approximately an order of magnitude less than the 10 year average life-span (Supplementary Table 2) of an adult worm. Consequently, perturbations in the proportion of adult worms in the various modelled states are strongly damped. Therefore, seasonal variations in the FOI—which are probably far less extreme that those considered in this sensitivity test—would have a negligible impact on the estimated parameter posterior distributions and on the overall adequacy of fit of the model.


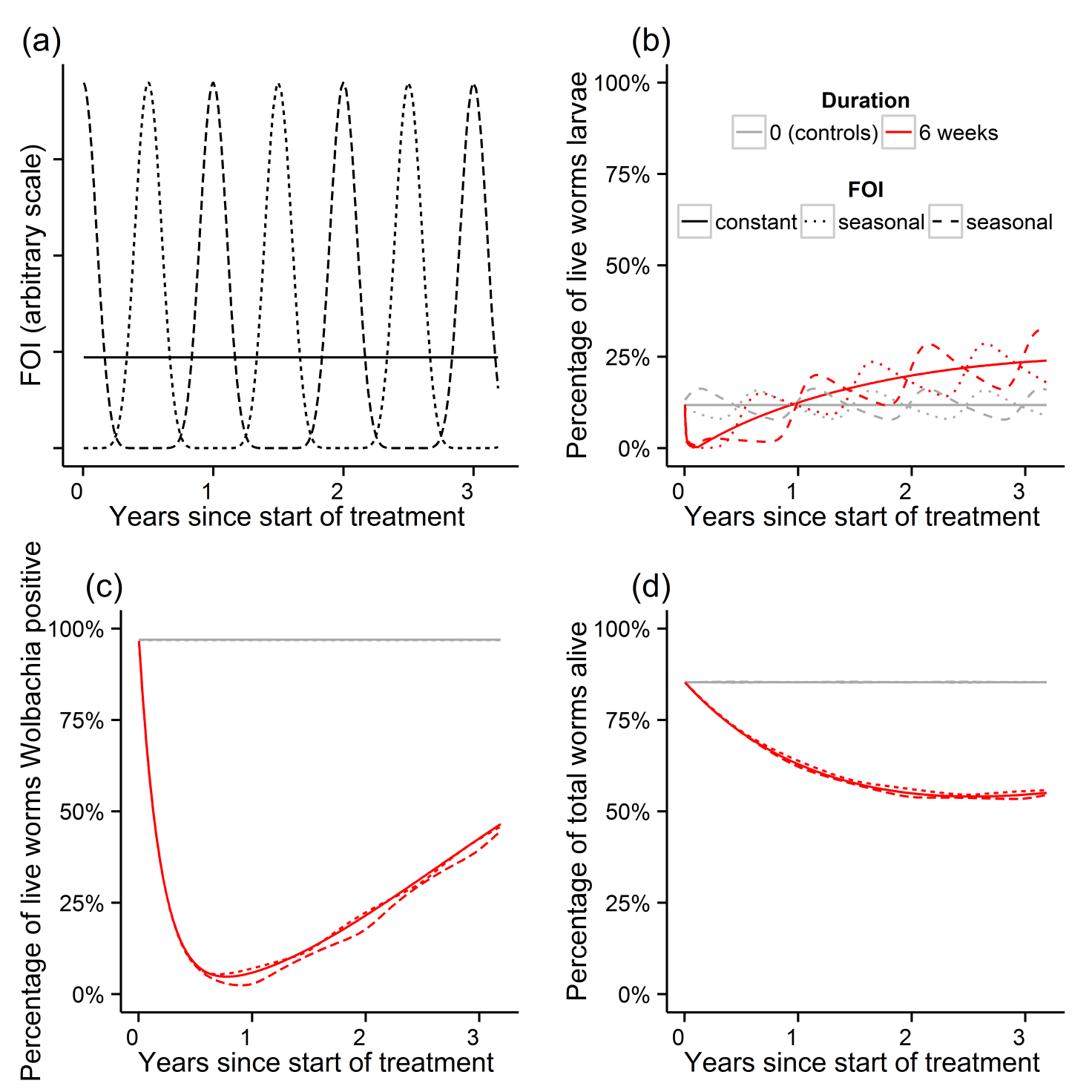


## Supplementary Figure 3. Sensitivity of model predictions to seasonal variation in the force of infection. In each panel the solid line represents a constant force of infection (FOI) and the broken (dashed and dotted) lines represent a seasonal pattern in the FOI such that transmission of *Onchocerca volvulus* worms occurs only during a rainy season which lasts for four to five months. The dashed and dotted lines correspond to, respectively, scenarios where the peak and nadir of the FOI occur at the start of doxycycline treatment. Panel (a) depicts the sinusoidal oscillations in the FOI over time since the start of doxycycline treatment, over the range of observed follow-up times (up to about 3 years and 3 months after the start of treatment with doxycycline, see Supplementary Table 1). Panels (b), (c) and (d) depict, respectively, the percentage of live worms in the pre-adult larval state, the percentage of live worms *Wolbachia-*positive and the percentage of total worms (dead and alive) that are alive with time after the start of doxycycline treatment. In each of these panels, the grey and red lines line correspond to, respectively, an ‘average’ patient (i.e. ignoring extraneous variation among individual patients) either untreated (or placebo treated) or given a 6 week course of 200mg doxycycline per day.

## Model structural uncertainty

The population dynamics model considers female *O. volvulus* as being in one of 5 states: larval, *Wolbachia-*positive, *Wolbachia-*depleted, *Wolbachia-*negative and dead (see Figure 1, main text). Each state is modelled using a single compartment, implying that—in the stochastic model analogue—the distribution of transition times between contiguous compartments is exponential [40]. This assumption is ubiquitous in infectious disease modelling in general [41] and in models of onchcoerciasis in particular [42, 43]. However, in the context of microparasitic infections, the substantive impact of this assumption on epidemiologically important quantities (notably the basic reproduction number *R*0) estimated by fitting such models to data has been highlighted [44, 45].

In a compartmental model framework, alternatives to the exponential distribution of transition times can be modelled by introducing additional latent compartments between contiguous states [45] (see Figure 1, main text). For example, including *l* latent compartments between the *Wolbachia-*depleted and *Wolbachia-*negative states and setting the rate of progression between the now multiple *Wolbachia-*depleted compartments to (*l*+ 1) × ζ, yields a distribution of transition times between the *Wolbachia-*depleted state and the *Wolbachia-*negative state which is gamma rather than exponential. Specifically, the distribution is gamma with mean (*l +*1) / ζ and precision (*l* + 1) ζ^2^. Hence, in the special case where *l* = 0, the distribution is exponential with mean 1 / ζ and precision ζ^2^ [40].

We explored the effect of introducing latent compartments on the estimated parameter posteriors and the accompanying derived efficacies of the various doxycycline treatment regimens. We refitted the model using alternative structural configurations, including either multiple (1, 2, or 4) latent compartments between the *Wolbachia-*depleted and *Wolbachia-*negative states, referred to as latent *Wolbachia-*depleted compartments, or between the various live compartments (*Wolbachia-*positive, -depleted and -negative) and the dead compartment, referred to as latent live compartments’. The introduction of latent live compartments allows the adult worm life span to be gamma rather than exponentially distributed.

The various alternative structural configurations all produced visually very similar fits to the observed data. However, deviance information criteria (DIC) [46] (Supplementary Table 3) indicated that the inclusion of at least one latent compartment, either *Wolbachia*-depleted or live, generally improved the fit of the model. However, estimated efficacies were largely invariant to the model’s structural configuration; the percentage change in the marginal efficacy rose by less than 4% in all cases (Supplementary Figure 4). By contrast, some of the (marginal) parameter posteriors were influenced by the model’s structural configuration (Supplementary Figure 5). In particular, introducing and then increasing the number of live latent compartments to 4 increased the estimated average life-span of worms either *Wolbachia*-depleted or *Wolbachia*-positive by 38% (Supplementary Figure 5) from a posterior mean of 2.1 years (1.7 to 2.7, 95% Bayesian credible interval, BCI) (see Table 2, main text) to 3.0 (2.5 to 3.5 95% BCI). Similarly, the estimated average clearance time of *Wolbachia* populations from adult worms rose by approximately 24% (Supplementary Figure 5) from a posterior mean of 70 days (56 to 85, 95% BCI) to 87 days (74 to 99, 95% BCI) when the number of *Wolbachia-*depleted latent compartments was increased from 0 to 4.

| Supplementary Table 3. Deviance information criteria (DIC) of models with different structural configurations | | | |
| --- | --- | --- | --- |
| Number of live latent compartments | Number of *Wolbachia* depleted latent compartments | DIC | ΔDIC^a^ |
| 0 | 0 | 1154 | ±0 |
| 1 | 0 | 1149 | −5 |
| 2 | 0 | 1146 | −8 |
| 4 | 0 | 1146 | −8 |
| 0 | 1 | 1149 | −5 |
| 0 | 2 | 1149 | −5 |
| 0 | 4 | 1157 | +1 |

^a^ ^b^ Change in the DIC compared to the model without latent compartments

In principle it might be possible to determine the optimum model structure by comparing DIC (or other information criteria) among a judiciously selected subset of an infinite number of possible model configurations. In practice, this task would be complicated by: (a) changes in the likelihood becoming increasingly negligible for increasing numbers of additional latent compartments, and (b) the computational demands of numerically solving differential equations with many state variables (i.e. compartments), potentially multiple times at each MCMC iteration. Alternatively, a more comprehensive approach would involve incorporating structural parameters which dynamically alter the structure of the compartmental model throughout the fitting process. We are not aware of any studies that have developed such an approach although it is known that—outside of the Bayesian paradigm—even solving optimization problems (i.e. maximising a log-likelihood) comprising a mixture of discrete and continuous variables (structural and rate parameters respectively) is a complex task (see [47] and references therein) and far beyond the scope of this work.


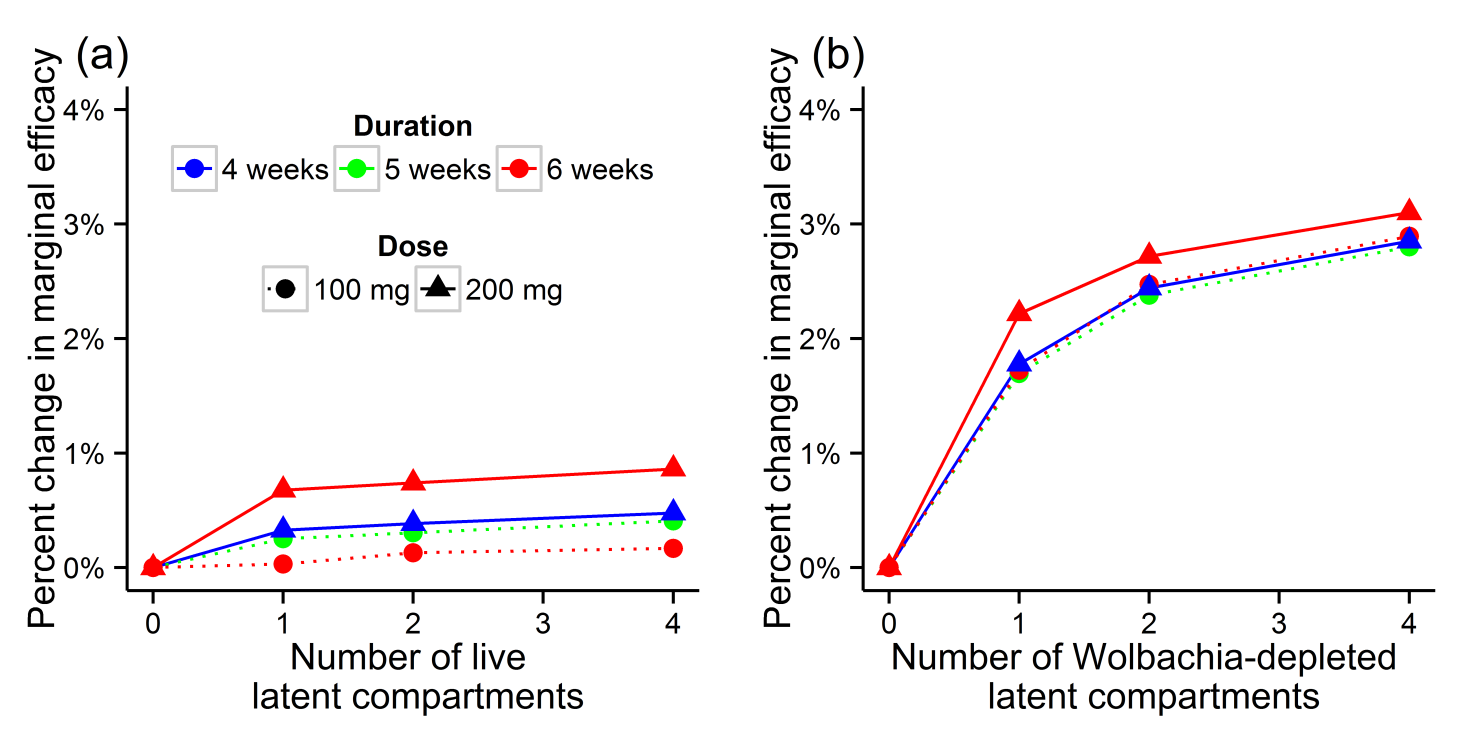


## Supplementary Figure 4. Sensitivity estimated efficacies to the structural configuration of the model. The percentage change in the posterior of the marginal efficacy (averaged over individual patients) estimated for each trialled drug regimen is plotted against the number of live (a) or *Wolbachia-*depleted (b) latent compartments included in the structurally modified model. Drug regimens are indicated as follows: 100 mg daily for 5 weeks, green circles joined by green dotted lines; 100 mg daily for 6 weeks, red circles joined by red dotted lines, 200 mg daily for 4 weeks, blue triangles joined by solid lines; 200 mg daily for 6 weeks, red triangles joined by solid lines.

# Supplementary References


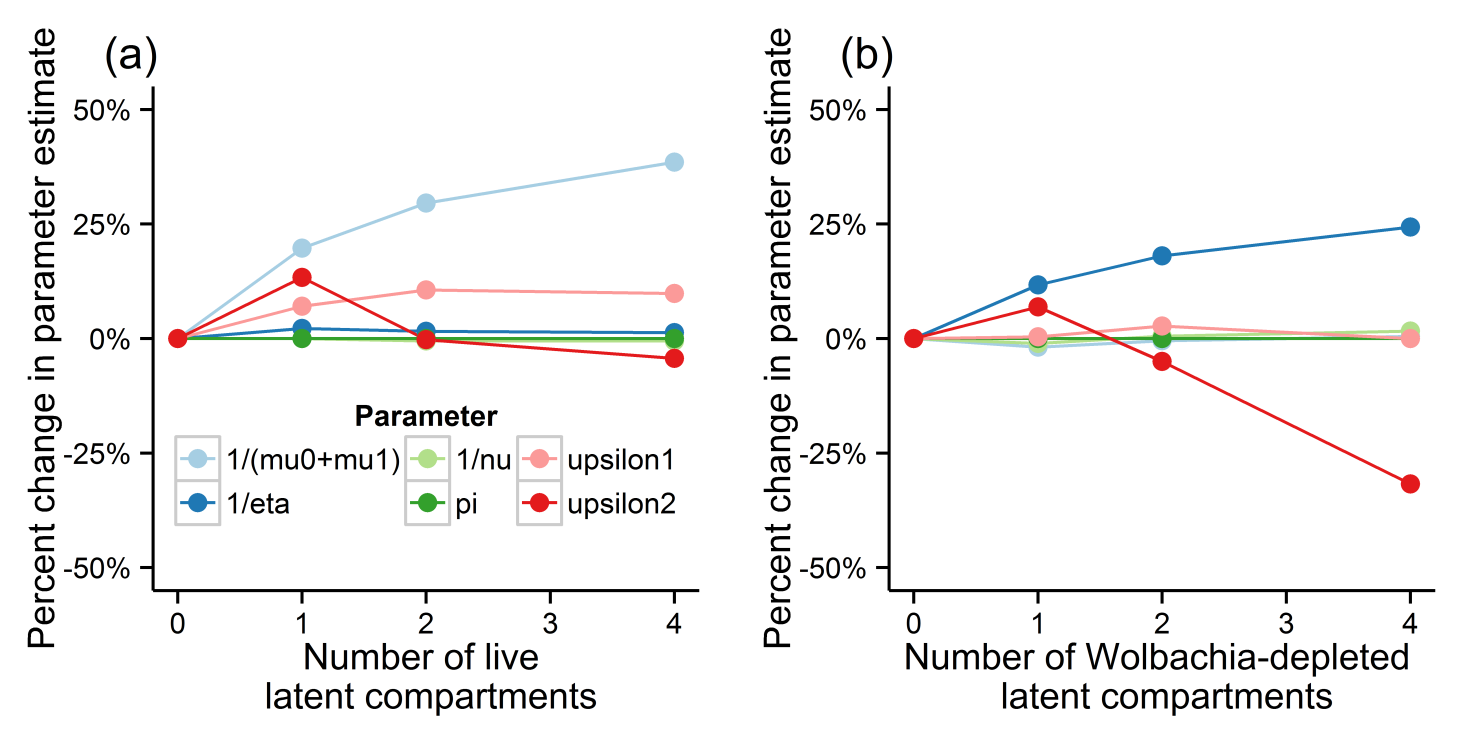


## Supplementary Figure 5. Sensitivity of estimated parameters to the structural configuration of the model. The percentage change in the posterior mean of the parameter estimates is plotted against the number of live (a) or *Wolbachia-*depleted (b) latent compartments included in the structurally modified model. Parameters are indicated as follows: life-expectancy of *Wolbachia-*depleted and *Wolbachia*-negative worms [1/(*µ*_0_ + *µ*_1_)], light blue circles joined by light blue lines; the average clearance time of *Wolbachia* populations from adult worms (1/*ζ*), dark blue circles joined by dark blue lines; average resorption time of dead adult worms (1/*η*), light green circles joined by light green lines; probability that a worm is correctly identified as *Wolbachia-*positive (*π*), dark green circles joined by dark green lines; inverse variance (= precision) among individual patients in the proportion of live female worms (*υ*_1_), light red/pink circles joined by light red/pink lines; inverse variance (= precision) among individual patients in the proportion of *Wolbachia* positive female worms (*υ*_2_), dark red circles joined by dark red lines.

1. Hoerauf A, Mand S, Volkmann L, et al. Doxycycline in the treatment of human onchocerciasis: kinetics of *Wolbachia* endobacteria reduction and of inhibition of embryogenesis in female *Onchocerca* worms. Microb Infect **2003**; 5(4): 261-73.

2. Hoerauf A, Specht S, Büttner M, et al. *Wolbachia* endobacteria depletion by doxycycline as antifilarial therapy has macrofilaricidal activity in onchocerciasis: a randomized placebo-controlled study. Med Microbiol Immunol **2008**; 197(3): 295-311.

3. Hoerauf A, Specht S, Marfo-Debrekyei Y, et al. Efficacy of 5-week doxycycline treatment on adult *Onchocerca volvulus*. Parasitol Res **2009**; 104(2): 437-47.

4. Hoerauf A, Volkmann L, Hamelmann C, et al. Endosymbiotic bacteria in worms as targets for a novel chemotherapy in filariasis. Lancet **2000**; 355(9211): 1242-3.

5. Specht S, Hoerauf A, Adjei O, Debrah A, Büttner DW. Newly acquired *Onchocerca volvulus* filariae after doxycycline treatment. Parasitol Res **2009**; 106(1): 23-31.

6. Tamarozzi F, Tendongfor N, Enyong PA, et al. Long term impact of large scale community-directed delivery of doxycycline for the treatment of onchocerciasis. Parasit Vectors **2012**; 5: 53.

7. Wanji S, Tendongfor N, Nji T, et al. Community-directed delivery of doxycycline for the treatment of onchocerciasis in areas of co-endemicity with loiasis in Cameroon. Parasit Vectors **2009**; 2(1): 39.

8. Turner JD, Tendongfor N, Esum M, et al. Macrofilaricidal activity after doxycycline only treatment of *Onchocerca volvulus* in an area of *Loa loa* co-endemicity: a randomized controlled trial. PLoS Negl Trop Dis **2010**; 4(4): e660.

9. Czock D, Markert C, Hartman B, Keller F. Pharmacokinetics and pharmacodynamics of antimicrobial drugs. Expert Opin Drug Metab Toxicol **2009**; 5(5): 475-87.

10. Rowland M, Tozer TN. Clinical Pharmacokinetics, Concepts and Applications. Philadelphia: Lippincott Williams and Wilkins, **1995**.

11. Austin DJ, White NJ, Anderson RM. The dynamics of drug action on the within-host population growth of infectious agents: melding pharmacokinetics with pathogen population dynamics. J Theor Biol **1998**; 194(3): 313-39.

12. Drusano GL. Antimicrobial pharmacodynamics: critical interactions of 'bug and drug'. Nat Rev Microbiol **2004**; 2(4): 289-300.

13. Duke BOL. The population-dynamics of *Onchocerca volvulus* in the human host. Trop Med Parasitol **1993**; 44(2): 61-8.

14. Agwuh KN, MacGowan A. Pharmacokinetics and pharmacodynamics of the tetracyclines including glycylcyclines. J Antimicrob Chemother **2006**; 58(2): 256-65.

15. Barbour A, Scaglione F, Derendorf H. Class-dependent relevance of tissue distribution in the interpretation of anti-infective pharmacokinetic/pharmacodynamic indices. Int J Antimicrob Agents **2010**; 35(5): 431-8.

16. Goutelle S, Maurin M, Rougier F, et al. The Hill equation: a review of its capabilities in pharmacological modelling. Fundam Clin Pharmacol **2008**; 22(6): 633-48.

17. Ferri E, Bain O, Barbuto M, et al. New insights into the evolution of *Wolbachia* infections in filarial nematodes inferred from a large range of screened species. PLoS One **2011**; 6(6): e20843.

18. Hermans PG, Hart CA, Trees AJ. In vitro activity of antimicrobial agents against the endosymbiont *Wolbachia pipientis*. J Antimicrob Chemother **2001**; 47(5): 659-63.

19. Fenollar F, Maurin M, Raoult D. *Wolbachia pipientis* growth kinetics and susceptibilities to 13 antibiotics determined by immunofluorescence staining and real-time PCR. Antimicrob Agents Chemother **2003**; 47(5): 1665-71.

20. Rolain JM, Maurin M, Vestris G, Raoult D. In vitro susceptibilities of 27 rickettsiae to 13 antimicrobials. Antimicrob Agents Chemother **1998**; 42(7): 1537-41.

21. Rolain JM, Stuhl L, Maurin M, Raoult D. Evaluation of antibiotic susceptibilities of three rickettsial species including *Rickettsia felis* by a quantitative PCR DNA assay. Antimicrob Agents Chemother **2002**; 46(9): 2747-51.

22. Duerr HP, Dietz K, Schulz-Key H, Büttner DW, Eichner M. Density-dependent parasite establishment suggests infection-associated immunosuppression as an important mechanism for parasite density regulation in onchocerciasis. Trans R Soc Trop Med Hyg **2003**; 97(2): 242-50.

23. Basáñez M-G, Collins RC, Porter CH, Little MP, Brandling-Bennett D. Transmission intensity and the patterns of *Onchocerca volvulus* infection in human communities. Am J Trop Med Hyg **2002**; 67(6): 669-79.

24. Albiez EJ. Calcification in adult *Onchocerca volvulus*. Trop Med Parasitol **1985**; 36(3): 180-1.

25. Diggle PJ, Heagerty P, Liang K-Y, Zeger SL. Analysis of Longitudinal Data. 2nd ed. Oxford: Oxford University Press, **2002**.

26. Gelman A, Carlin JB, Stern HS, Rubin DB. Bayesian Data Analysis. 2^nd^ ed. London: Chapman & Hall, **2004**.

27. Haario H, Saksman E, Tamminen J. An adaptive Metropolis algorithm. Bernoulli **2001**; 7: 223-42.

28. Roberts GO, Rosenthal JS. Examples of adaptive MCMC. J Comput Graph Stat **2009**; 18: 349-67.

29. R Development Core Team. R: A language and environment for statistical computing. Vienna: R Foundation for Statistical Computing, **2011**.

30. Soetaert K, Petzoldt T, Setzer RW. Solving differential equations in R: package deSolve. J Stat Soft **2010**; 33: 1-25.

31. Albert J, Chib S. Bayesian modeling of binary repeated measures data with application to corssover trials. In: Berry DA, Stangel DK. Bayesian Biostatistics. New York: Marcel Dekker, **1996**: 577-99.

32. Chib S, Carlin BP. On MCMC sampling in hierarchical longitudinal models. Stat Comput **1999**; 9: 17-26.

33. Eddelbuettel D. Seamless R and C++ Integration with Rcpp. New York: Springer, **2013**.

34. Cheke RA, Sowah SA, Avissey HSK, Fiasorgbor GK, Garms R. Seasonal variaion in onchocerciasis tranmssion by *Simulium squamosum* at perennial breeding sites in Togo. Trans R Soc Trop Med Hyg **1992**, 86: 67-71.

35. Opara KN, Fagbemi OB, Ekwe A, Okenu DM. Status of forest onchocerciasis in the Lower Cross River basin, Nigeria: entomologic profile after five years of ivermectin intervention. Am J Trop Med Hyg **2005**, 73(2): 371-6.

36. Griffen JT, Hollingsworth TD, Okell LC, et al. Reducing *Plasmodium falciparum* malaria transmission in Africa: A model-based evaluation of intervention strategies. PLoS Med **2010,** 7(8): e1000234.

37. Diawara L, Diawara L, Traoré MO, et al. Feasibility of onchocerciasis elimination with ivermectin treatment in endemic foci in Africa: first evidence from studies in Mali and Senegal. PLoS Negl Trop Dis **2009**; 3(7): e497.

38. Traoré MO, Sarr MD, Badji A, et al. Proof-of-principle of onchocerciasis elimination with ivermectin treatment in endemic foci in Africa: final results of a study in Mali and Senegal. PLoS Negl Trop Dis. **2012**, 6(9): e1825.

39 Tekle A, Elhassan E, Isiyaku S, et al. Impact of long-term treatment of onchocerciasis with ivermectin in Kaduna State, Nigeria: first evidence of the potential for elimination in the operational area of the African Programme for Onchocerciasis Control. Parasit Vectors. **2012**, 5(1): 28.

40. Cox DR, Miller HD. The Theory of Stochastic Processes. London: Chapman & Hall, **1977**.

41. Anderson RM, May RM. Infectious Diseases of Humans: Dynamics and Control. Oxford: Oxford University Press, **1991**.

42. Basáñez MG, Boussinesq M. Population biology of human onchocerciasis. Phil Trans R Soc Lond B **1999**; 354: 809-26.

43. Filipe JA, Boussinesq M, Renz A, et al. Human infection patterns and heterogeneous exposure in river blindness. Proc Nat Acad Sci USA **2005**; 102: 15265-70.

44. Wallinga J, Lipsitch M. How generation intervals shape the relationship between growth rates and reproduction numbers. Proc Biol Sci **2007**; 274(1609): 599-604.

45. Wearing HJ, Rohani P, Keeling MJ. Appropriate models for the mangement of infectious diseases. PLoS Med **2005**; 2: e174.

46. Spiegelhalter DJ, Best NG, Carlin BR, van der Linde A. Bayesian measures of model complexity and fit. J Roy Stat Soc B **2002**; 64: 583-616.

47. Nocedal J, Wright SJ. Numerical Optimization. New York: Springer, **2000**.

48. Prost A. Pre-patent period in onchocerciasis. Bull World Health Organ **1980**; 58(6): 923-5.

49. Plaisier AP, van Oortmarssen GJ, Remme J, Habbema JD. The reproductive lifespan of *Onchocerca volvulus* in West African savanna. Acta Trop **1991**; 48(4): 271-84.

50. Welling PG, Koch PA, Lau CC, Craig WA. Bioavailability of tetracycline and doxycycline in fasted and nonfasted subjects. Antimicrob Agents Chemother **1977**; 11(3): 462-9.

51. Saivin S, Houin G. Clinical pharmacokinetics of doxycyline and minocycline. Clin Pharmacokinet **1988**; 15(6): 355-66
